# COVID-19 Vaccine Hesitancy in the United States: A Systematic Review

**DOI:** 10.3389/fpubh.2021.770985

**Published:** 2021-11-23

**Authors:** Farah Yasmin, Hala Najeeb, Abdul Moeed, Unaiza Naeem, Muhammad Sohaib Asghar, Najeeb Ullah Chughtai, Zohaib Yousaf, Binyam Tariku Seboka, Irfan Ullah, Chung-Ying Lin, Amir H. Pakpour

**Affiliations:** ^1^Department of Internal Medicine, Dow University of Health Sciences, Karachi, Pakistan; ^2^Department of Internal Medicine, Dow University Ojha Hospital, Karachi, Pakistan; ^3^Department of General Surgery, Liaquat National Hospital and Medical College, Karachi, Pakistan; ^4^Department of Internal Medicine, Hamad Medical Corporation, Doha, Qatar; ^5^Department of Public Health, Dilla University, Dilla, Ethiopia; ^6^Department of Community Medicine, Kabir Medical College, Peshawar, Pakistan; ^7^Institute of Allied Health Sciences, National Cheng Kung University Hospital, College of Medicine, National Cheng Kung University, Tainan, Taiwan; ^8^Department of Nursing, School of Health and Welfare, Jönköping University, Jönköping, Sweden

**Keywords:** COVID-19 vaccines, severe acute respiratory coronavirus 2 (SARS-CoV-2), vaccine hesitancy, vaccine acceptance, United States, intent to vaccinate

## Abstract

Vaccine hesitancy in the US throughout the pandemic has revealed inconsistent results. This systematic review has compared COVID-19 vaccine uptake across US and investigated predictors of vaccine hesitancy and acceptance across different groups. A search of PUBMED database was conducted till 17th July, 2021. Articles that met the inclusion criteria were screened and 65 studies were selected for a quantitative analysis. The overall vaccine acceptance rate ranged from 12 to 91.4%, the willingness of studies using the 10-point scale ranged from 3.58 to 5.12. Increased unwillingness toward COVID-19 vaccine and Black/African Americans were found to be correlated. Sex, race, age, education level, and income status were identified as determining factors of having a low or high COVID-19 vaccine uptake. A change in vaccine acceptance in the US population was observed in two studies, an increase of 10.8 and 7.4%, respectively, between 2020 and 2021. Our results confirm that hesitancy exists in the US population, highest in Black/African Americans, pregnant or breastfeeding women, and low in the male sex. It is imperative for regulatory bodies to acknowledge these statistics and consequently, exert efforts to mitigate the burden of unvaccinated individuals and revise vaccine delivery plans, according to different vulnerable subgroups, across the country.

## Introduction

Vaccines are critical in lowering disease-specific mortality rates (1) and the long-standing control of the COVID-19 pandemic is pivoted upon the development and uptake of the vaccine (2). Vaccines currently recommended and authorized in the US are BNT162b2 (Pfizer-BioNTech), mRNA-1273 (Moderna), Johnson & Johnson/Janssen (3) with the challenge now shifting from finding an effective cure to ensuring its implementation (4).

The US Centers for Disease Control and Prevention acclaims vaccination as one of the leading success stories of public health in the twentieth century (1) and a similar feat is now being aimed for the novel coronavirus. As part of Operation Warp Speed, the US administration alongside manufacturers and developers exerted efforts and by January 2021, hoped to deliver 300 million doses of a safe and effective vaccine for COVID-19 (5). By mid-January 2021, there were around 13 million persons who had COVID-19 vaccine initiated in the US (6) and while these numbers were promising, the vaccination process has been met with an undesirable, although not unusual, phenomenon well-known as “vaccine hesitancy.” Defined by the World Health Organization as “the delay in the acceptance or refusal to vaccinate despite the availability of vaccine services” (7), the term vaccine “hesitant” is preferred over “pro” and “anti” vaccination to avoid polarization and, as it insinuates that the minds can be persuaded toward acceptance (8).

Since the inception of vaccines in medical practice in the 1800s (9), the public has not always concurred to getting vaccinated albeit many people have complied in face of apparent death and indisposition (1). Frequent outbreak of vaccine-preventable diseases in the US, such as 1,282 confirmed cases of measles in the United States in 2019 (8), can be ascribed to reduced vaccination and hence vaccine hesitancy, the latter ranked by the WHO as the top threat to global health in 2019 (1).

According to a global study, 72% of people would take the COVID-19 vaccine if deemed safe and effective, but willingness varies between countries (10). When studied under the framework of the 5C model of psychological antecedents that drive vaccine acceptance: confidence, constraints, complacency, calculation, and collective responsibility, the US population indicated 54% vaccine acceptance, a value that divulges vaccine skepticism (11). The US population has demonstrated inconsistent results over the period as between April 1–14 and November 25–December 8, 2020, the percentage who stated they were somewhat or very likely to get vaccinated declined from 74 to 56% (12). The percentage of US adults intending to get vaccinated has seen a u-shaped pattern with results showing changes between September and December 2020, which correspond to pre-authorization and post-authorization dates in the US, respectively (7). Conversely, another national representative survey revealed the vaccine hesitancy to have a longitudinal decline of 10.8% points between October 2020 and March 2021 (13).

COVID-19 vaccine receptivity in the US has varied between states and subgroups. In mid-October 2020, acceptance rates ranged from 38% in the Northeast to 49% in the West (14). Vaccine hesitancy in the general population has been correlated with certain factors including gender, age, race, socioeconomic status, education level, and US-based surveys disclose important findings. Women have lower intentions than men to be vaccinated (14) and as of a study from April to December 2020, the self-reported likelihood of getting COVID-19 vaccination was lower among females than males (51 vs. 62%) (12).

The cohort comprising of vaccine-hesitant individuals can be large enough to diminish the COVID-19 vaccine's potential to provide population immunity (15). Impediments to vaccination involve concerns including fear of side effects, inadequate information, short duration of immunity (14) forgoing vaccination due to lack of insurance or financial resources (16). Safety and effectiveness of the vaccine are the most pivotal detriments of hesitancy (4) while for some, especially marginalized factions, dissatisfaction with the health system owing to past experiences of discrimination, systematic racism deters them from vaccination (17).

Vaccine hesitancy is not a singular problem but attributed to various underlying causes that differ across time and communities (1). Recent results have shown somewhat reduced hesitancy, corresponding to the dates of vaccine approval and mass roll-out (13) and it is speculated that as the pandemic becomes more “real” to the Americans, vaccine acceptance can improve. Being one of the representative countries hardest hit by COVID-19, estimating vaccine hesitancy in the U.S could be important for future vaccine promotion and herd immunity (18). This systematic review aims to broaden the scope of discussion by studying factors coupled with vaccine hesitancy in the US population and the study's findings will be beneficial not only for COVID-19 vaccination coverage but also improving the existing healthcare system's preparedness for routine and emergency vaccination. Furthermore, our results will be imperative in helping strategize policies for tackling antagonism and for developing a thorough vaccine delivery plan.

## Methods

The review was performed following PRISMA guidelines. Papers published in MEDLINE (PubMed), Cochrane library, and Google Scholar assessing COVID-19 vaccine hesitancy/vaccine uptake/ vaccine acceptance in the English language were eligible for inclusion in the review. The inclusion criteria were: (1) peer-reviewed published articles indexed in PubMed; (2) survey studies among the general population, healthcare workers, minority and religious communities, students, or patients (3) the major aim of the study was to evaluate COVID-19 vaccine acceptance/uptake/hesitancy in the US population only and (4) publication language was English. The exclusion criteria were: (1) the article did not aim to evaluate COVID-19 vaccine acceptance/hesitancy/uptake in the US population; (2) publication language was not English.

A search was carried out till 17th July 2021 using the following search strategy: (COVID ^*^ vaccine ^*^ hesitancy [Title/Abstract]) OR (COVID ^*^ vaccine acceptance [Title/Abstract])) OR (COVID ^*^ vaccine ^*^ hesitancy [Title/Abstract])) OR (COVID ^*^ intention to vaccinate ^*^ [Title/Abstract]) OR (COVID vaccine ^*^ accept ^*^[Title/Abstract]).

Articles were screened by abstracts and titles. Studies shortlisted were cohort studies and cross-sectional studies that assessed COVID-19 vaccine acceptance over a certain time period, specific for each study, respectively. Studies drew comparison between vaccine acceptance and vaccine hesitance in the population surveyed. After selection, data extraction for the following items was conducted: title and date of the study, study period/duration, the target population of the study (e.g., general public, students, healthcare workers, patients, religious groups, and minority groups), region of US where the study took place, population characteristics i.e., sample size, % female, mean age, % Whites, the definition of vaccine acceptance in the study, overall acceptance rate, acceptance rate by education level, factors relating to vaccine acceptance, and factors relating to vaccine hesitancy. Overall vaccine acceptance (%) was taken from each study as deduced, corresponding to the definition of vaccine acceptance presented in the study. A forest plot was also constructed using Microsoft Excel version 2018 to demonstrate the overall prevalence of COVID-19 vaccine acceptance in the United States.

## Results

**Figure 1:**
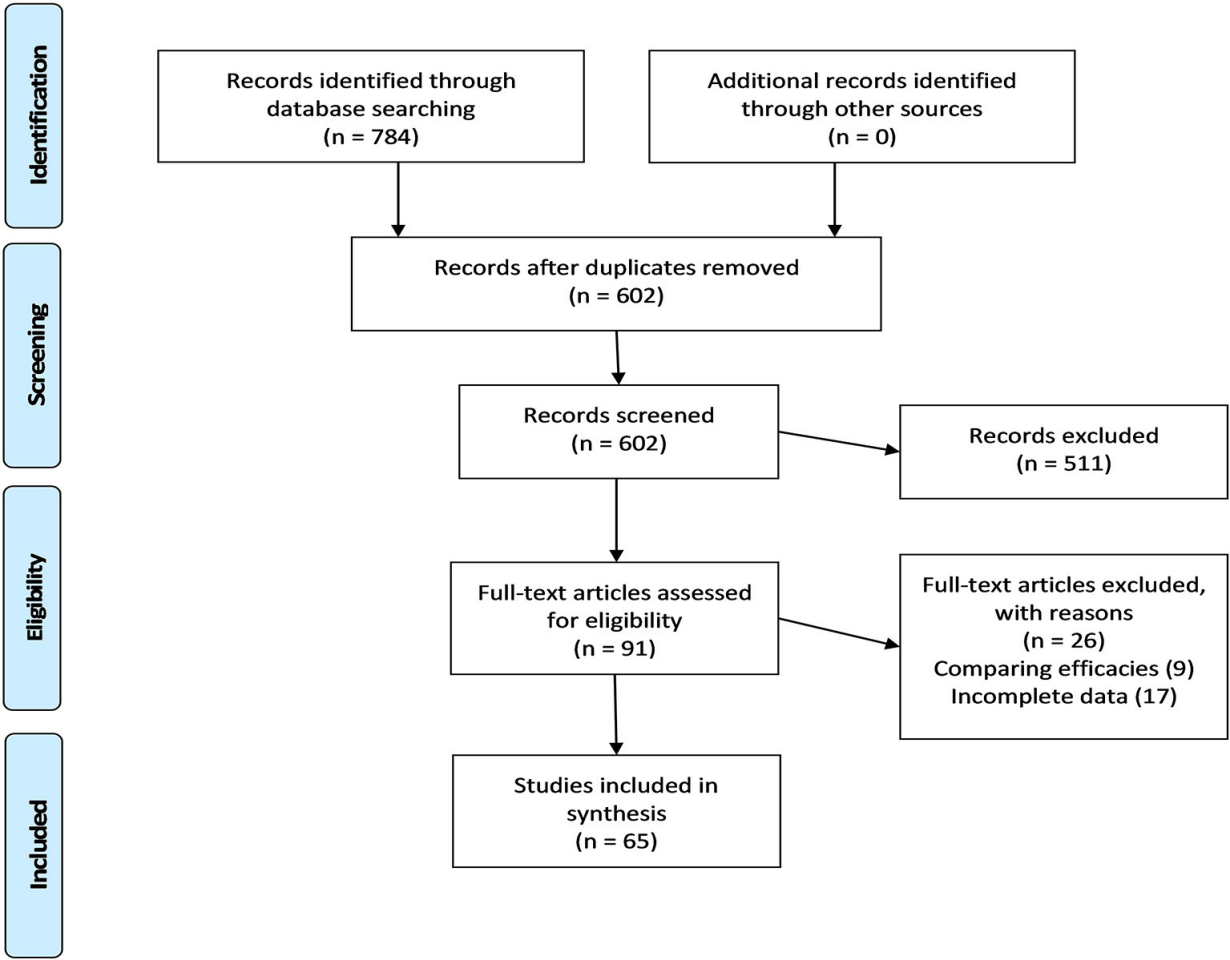
PRISMA guidelines flow chart.

The search string provided in the Supplementary Material retrieved 784 records, of which 65 were included in the quantitative synthesis of observational studies on vaccine acceptance in the United States (US). Figure 1 illustrates the four-step selection process as per the PRISMA guidelines (19).

### Characteristics of the Literature

The sample size of the selected studies ranged from 25 to 73,650, with a total of 3,13,998 individuals. Out of the 65 studies (67 surveys), 31 of them reported the study region, of which nine were conducted in New York City (NYC). Of the 50 studies reporting female sex %, 38 of them had a percentage >50. Acceptance was measured as somewhat likely, extremely likely, and willingness to take the vaccine. The majority of the surveys that reported race and ethnicity, had a predominant non-Hispanic white population. Vaccine acceptance was presented as percentages in 61 studies, whereas the remaining four studies reported means. Four of the studies used a point scale system to measure the likelihood of receiving the COVID-19 vaccine. Silva et al. (20) made use of a 10-point scale ranging from 1- extremely unlikely and 10- extremely likely, whereas Meier et al. (21), Rhodes et al. (22), and Dorman et al. (7) used a 7-point scale. Detailed studies characteristics are present in Table 1.

**Table 1:** Characteristics of the literature.

**Study**	**Study period**	**Population type/Settings**	**Region**	**Sample size (*N*)**	**Female sex %**	**White population %**	**Acceptance rate %**	**Predictors of hesitance**	**Predictors of acceptance**
Kobayashi et al. (23)	13th−16th November 2020	General	–	661	–	–	90.00%	–	–
Xiang et al. (24)	1st December−7th January 2021	Multiple sclerosis patients	Oregon and Southwest Washington	410	23.90%	89.50%	70.10%	Unsure about its safety, developed rapidly/possible side effects	–
Parente et al. (25)	14th August−28th August 2020	Employees or students at a medical centre	Kansas	3,292	78.00%	87.60%	90.10%	Safety, side effects, Black/African American	–
Garcia et al. (26)	8th January−11th February 2021	Dialysis center	–	1,515	43.00%	30.00%	72.30%	Black/African American, females	–
Thunstrom et al. (27)	March 2020	General	–	3,133	–	–	80.50%	Low trust in government agencies, females	–
Kecojevic et al. (28)	February–March 2021	Students	New Jersey	352	73.60%	49.50%	52.80%	–	Older age, non-Hispanic white, previous illness, and family member vaccinated
Yang et al. (29)	December–January 2021	Used tobacco or marijuana	–	387	53.50%	74.90%	49.10%	Black/African American	75.9% of willing receive flu vaccine
Carmody et al. (30)	7th December−20th January 2021	Orthodox Jewish	NYC	102	87.00%	–	12.00%	Natural infection to be better than vaccination for developing immunity	–
Stoler et al. (31)	4th−17th June, 2020	General	–	1,040	–	–	63.50%	Black/African American	Whites
Serper et al. (32)	13th−23rd December 2020	Chronic disease patients	Philadelphia, Pennsylvania	1,215	50.00%	86.50%	85.00%	Females, Black/African American, lower-income, and younger age	–
Willis et al. (15)	July–August 2020	General	Arkansas	1,205	75.23%	76.37%	78.14%	Black/African American	–
Lindholt et al. (11)	13th September 2020–16th February 2021	General	–	3,500	–	–	54.00%	–	–
Nikolovski et al. (33)	6th−20th November 2020	General	–	7,402	46.20%	91.80%	91.30%	Concerns about its safety and efficacy. Females, Black/African American	–
Thompson et al. (34)	June–December 2020	General	Michigan	1,835	79%	52%	48%	Black/African American	–
Ou et al. (35)	11th November−2nd December 2020	Solid organ transplant recipients and non-SOTRs	–	2,925	61.10%	73.20%	56.10%	Lead to organ transplant rejection	
Theis et al. (36)	November 2020–January 2021	Individuals associated with Wright-Patterson Air Force Base	Dayton, Ohio	816	–	–	77.33%	Side effects, and the spread of misinformation	–
Ciardi et al. (37)	10th December 2020–5th January 2021	Healthcare workers	The Bronx, New York City, NY	428	65%	24%	64%	–	–
Liu et al. (18)	29th January−13th February 2021	General	–	3,702	51.80%	–	72.20%	Efficacy of COVID-19 vaccines, cost of vaccination	–
Gatwood et al. (38)	3rd−18th June 2020	General	Tennessee	1,000	51%	80.10%	45.90%	Effectiveness, perceived lack of disease risk, and vaccine safety concerns	–
Tsapepas et al. (39)	March 2021	Kidney and pancreas transplant recipients in New York City	New York City, NY	664	42.77%	27.26%	31.63%	Younger age, Black/African American, medium level of poverty, safety in transplant recipients	–
Bass et al. (17)	15th May−6th July 2020	General	Miami, FL, New York City, NY, San Francisco, CA	501	36.00%	24.00%	87.00%	Younger age, females high school education, lower-income, Black/African American	–
Johnson et al. (40)	15th September−4th December 2020	General	Louisiana	248	57%	(Black 65.2%)	32.66%	Side effects, females, Black/African American, high school, older age	–
Daly et al. (13)	October 2020	General	–	6,016	-	-	54.00%	Younger age Females. Black/African American, Lower-income	–
Daly et al. (13)	1st March 2021	General	–	6,035	–	–	64.80%	Younger age, Females, Black/African American, Lower-income	–
Mascarenhas et al. (41)	2020	Dental students	Michigan, Florida, and Utah	248	58%	55.50%	83.70%	–	–
Marquez et al. (42)	28th September−10th November 2020	Caregivers whose children were being treated in the dental clinic at Cincinnati Children's Hospital Medical Center	Cincinnati, Ohio	99	83.50%	72.90%	60.80%	–	–
Sutton et al. (43)	7th−29th January 2021	All women at the Department of Obstetrics and Gynecology, Columbia University Medical Center	New York City, NY	1,012	100%	46.90%	61%	Pregnant	Non-pregnant
Kelekar et al. (44)	September–December 2020	3 US dental schools and 1 US medical school	Michigan, Florida, and Utah	415	–	–	63.85%	Dental students	Medical students
Chin et al. (45)	22nd December−4th March 2021	State prison	California	97,779	–	–	66.50%	Black/African American	–
Levy et al. (46)	14th December 2020–14th January 2021	Pregnant women	New York City, NY	662	100%	–	58.35%	Risk to fetus, side effects, Black/African American	Non-Hispanic white
Ruggiero et al. (47)	–	General	–	427	–	–	44.00%	Fear of vaccines, being against all forms of vaccines, religious reasons	–
Doherty et al. (preprint) (48)	27th August−15th December 2020	General	North Carolina	948	63	27.7%	68.90%	Black/African American, safety concerns	–
Silva et al. (49)	November 2020	Student at Health Services and College of Pharmacy attending influenza clinics	Rhode Island	237	65%	84%	50%	Safety, effectiveness, and limited information	–
Trent et al. (50)	July–August 2020	General	New York City, NY, and Phoenix, Arizona	1,704	53%	–	71% NY; 76% Phoenix	Younger age, females	–
Silva et al. (20)	19th October 16th December 2020	Sexual and Gender Minority Men and Transgender Women	–	1,350	–	25.90%	7 (3.12)	–	–
Hou et al. (51)	13th June−31st July 2020	Twitter posts mentioning COVID vaccine	New York City, NY	1,568	–	–	36.40%	–	–
Nguyen et al. (preprint) (52)	24th March 2020–16th February 2021	General	–	73,650	–	–	91.40%	Younger age, Females, Black/African American	–
Geana et al. (53)	5th−25th March 2021	Women recently released from jail	Midwest USA	25	100%	80.00%	20%	The pace of development, adverse effects	–
Piltch-Loeb et al. (54)	13th−23rd December 2020	General	–	2,650	46.10%	66%	19.70%	Black/African American, females	Non-Hispanic whites, Males
Meier et al. (21)	28th−30th October 2020	General	–	1,054	51.20%	75.20%	5.12 (1.98)	–	–
Latkin et al. (55)	March, May, July 2020	General	–	592	56%	79.40%	59.10%	–	Males
Scott et al. (56)	April 2020	Amish families	Ohio	391	–	–	25%	–	–
Szilagyi et al. (12)	25th November−8th December 2020	Uninstitutionalized US residents	–	5,660	–	–	56.2%	Female, Black/African American	Asian, Males, Older age
Szilagyi et al. (12)	1st−14th April 2021	Uninstitutionalized US residents	–	5,660	–	–	74.1%	Female, Black/African American	Asian, Males, Older age
Callaghan et al. (16)	28th May−8th June 2020	General	–	5,009	51.5	60%	68.90%	–	–
Mercadante and Law (57)	23rd−29th October 2020.	General	–	525	49%	66.10%	66.70%	–	–
Kociolek et al. (58)	21st December−13th January 2021	Children's hospital staff	Chicago, Illinois	4,448	81.60%		59.80%	Black/African American	–
Ruiz and Bell (59)	15th−16th June 2020	General	–	804	53.60%	65.3%	14.80%	–	Males, non-Hispanic white, democrats, college-educated
Latkin et al. (60)	14th−18th May 2020	General	–	1,043	70.10%	69.40%	53.60%	Black/African American	–
Keene Woods et al. (61)	April–May 2020	General	Kansas	53	96.15%	50.94%	59.60%	–	–
Greenhawt et al. (62)	April 2020	General	–	1,262	–	–	65.70%	–	–
Fisher et al. (63)	16th−20th April 2020	General	–	991	51.50%	63.3%	57.60%	Younger age, Black/African American, Females, lower educational attainment, not having received the influenza vaccine, need for more information, anti-vaccine attitudes or beliefs, lack of trust	–
Dorman et al. (7)	October–November 2020	General	–	26,324	72.80%	51.6%	4.6	Females or Hispanic or Black/African American	Males, older age, non-Hispanic whites or Asian
Khubchandani et al. (64)	June 2020	General	–	1,878	52%	74%	78%	Black/African American, Hispanics, lower education and incomes, rural dwellers, people in the northeastern US, Republicans	Asian, others
Kelly et al. (10)	April 2020	General	–	2,279	52%	78%	75%	Black/African American Females	Hispanics, non-Hispanic whites, males
Rungkitwattanakul et al. (65)	–	Patients receiving dialysis treatment	–	90	40%	(Black 83%)	49%	Lack of trust in the federal government Lack of information, safety do not trust the manufacturer of the vaccine	–
Kelkar et al. (66)	31st December 2020–8th January 2021	Cancer patients	Florida	205	79%	82%	71%	–	–
Malik et al. (67)	May 2020	General	–	672	57%	73%	67%	Females, Black/African American	Males, older age, non-Hispanic whites or Asian, college degree
Stern et al. (68)	September–December 2020	Residents in three prisons and 13 jails in four states	Washington, Florida, California, Texas	5,110	17.60%	41.90%	44.90%	Black/African American, Younger age, lived in jails vs. prisons waiting for more information, efficacy, or safety concerns	–
Pogue et al. (69)	–	General	–	316	49.38%	63.27%	68.54%	–	–
Salmon et al. (70)	25th November−7th December 2020	General	–	2,525	51.80%	39.70%	50%	Females, black/African American	Males, non-Hispanic whites
Unroe et al. (71)	14th−17th November 2020	Nursing home staff	Indiana	8,243	87%	82.60%	45%	Side effects, females, Black/African American	Males, non-Hispanic whites
Rhodes et al. (22)	2020	Vaccine hesitant parents	–	1,381	61.80%	68.80%	3.58 (2.16)	General unwillingness	More educated respondents
Lucia et al. (72)	–	Medical students	Southeast Michigan	167	57%	–	75.40%	–	–
Reiter et al. (73)	May, 2020	General	–	2,006	56%	67%	69%	Non-Latinx blacks	Recommended by a healthcare provider, effectiveness
Ehde et al. (74)	10th April−6th May 202	Multiple sclerosis patients	–	486	81.3%	90.5%	84.5%	–	More educated, greater risk of contracting
Nishma Research (75)	18th−28th April, 2021	Jewish Community	Majority from New York	3,666	42.4%	–	65.3%	Females, Less than college grad education, younger age	Males, older age, college graduate

### COVID-19 Vaccine Acceptance Rates

Vaccine acceptance rates varied from a low of 12% to a high of 91.4%. In 48 from 63 surveys, readiness to get vaccinated was ≥50%. The mean and standard deviation of acceptance rate by Silva et al. was 7 (3.12); meanwhile, the willingness of studies using the 10-point scale ranged from 3.58 to 5.12.

### COVID-19 Vaccine Acceptance Stratified per Region

**Figure 2:**
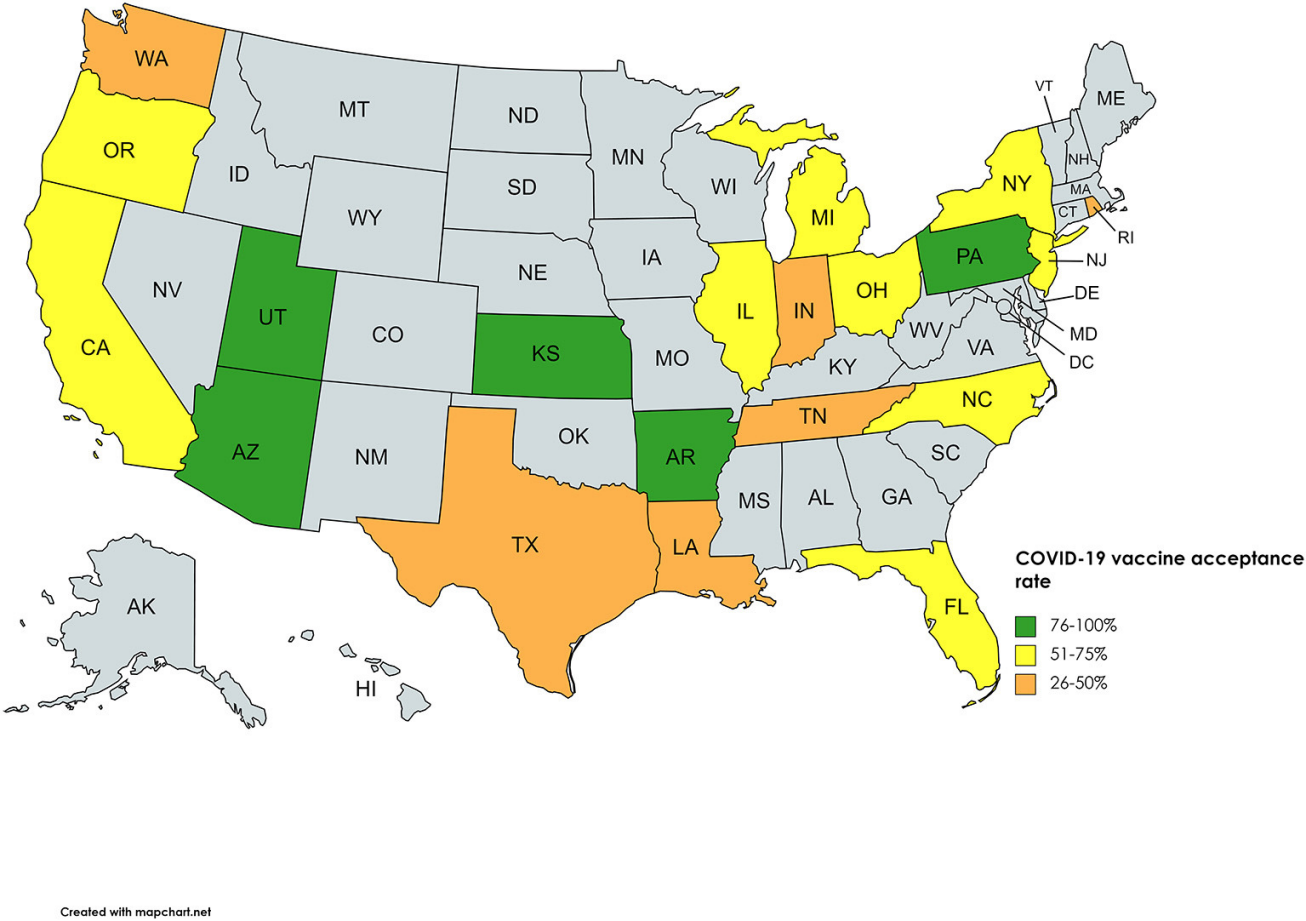
COVID-19 vaccine acceptance rates of United States.

A total of 32 studies included the geographical location of the survey, comprising data of 20 states out of a possible 50. Overall, a low of 12% was observed in New York City in the Orthodox Jewish community, whereas a high of 90.1% was reported in Kansas among employees and students at the University of Kansas Medical Centre. Figure 2 illustrates the geographical average COVID-19 vaccine acceptance percentage reported in each state. Classifying vaccine acceptance per the four regions in the United States shows New England division of Northeast, West North Central of Midwest, Mountain division of West, and the whole of the South, except West South Central, had a low percentage of states with vaccine acceptance figures, however, in the Mid Atlantic division of Northeast, all three states had surveys conducted, making it the only division with 100 percent data availability. Supplementary Table 1 states the percentage acceptance by each state, with Kansas reporting a high of 89.6%.

### COVID-19 Vaccine Acceptance Stratified per Respondents' Population

Apart from the studies with the general population, 18 surveys took place in a healthcare setting, including patients, workers and, students. From these 18, eight highlighted patients' attitudes toward the COVID-19 vaccine. Amongst patients, two surveys on solid organ transplant (SOT) recipients showed their hesitance toward the vaccine owing to lack of data and concerns of its safety in transplant recipients (56.10 and 31.63% acceptance). Other than SOT recipients, studies report willingness in patients with chronic disease, multiple sclerosis, dialysis, and cancer which showed a positive response toward the COVID-19 vaccine. Of these, patients suffering from chronic disease reported the highest acceptance rate of 85%. Healthcare workers and students (dental and medical) showed a varying attitude toward the vaccine as acceptance ranged from a low of 45% in nursing home staff to a high of 90.10% in staff and students at the University of Kansas Medical Centre.

**Figure 3:**
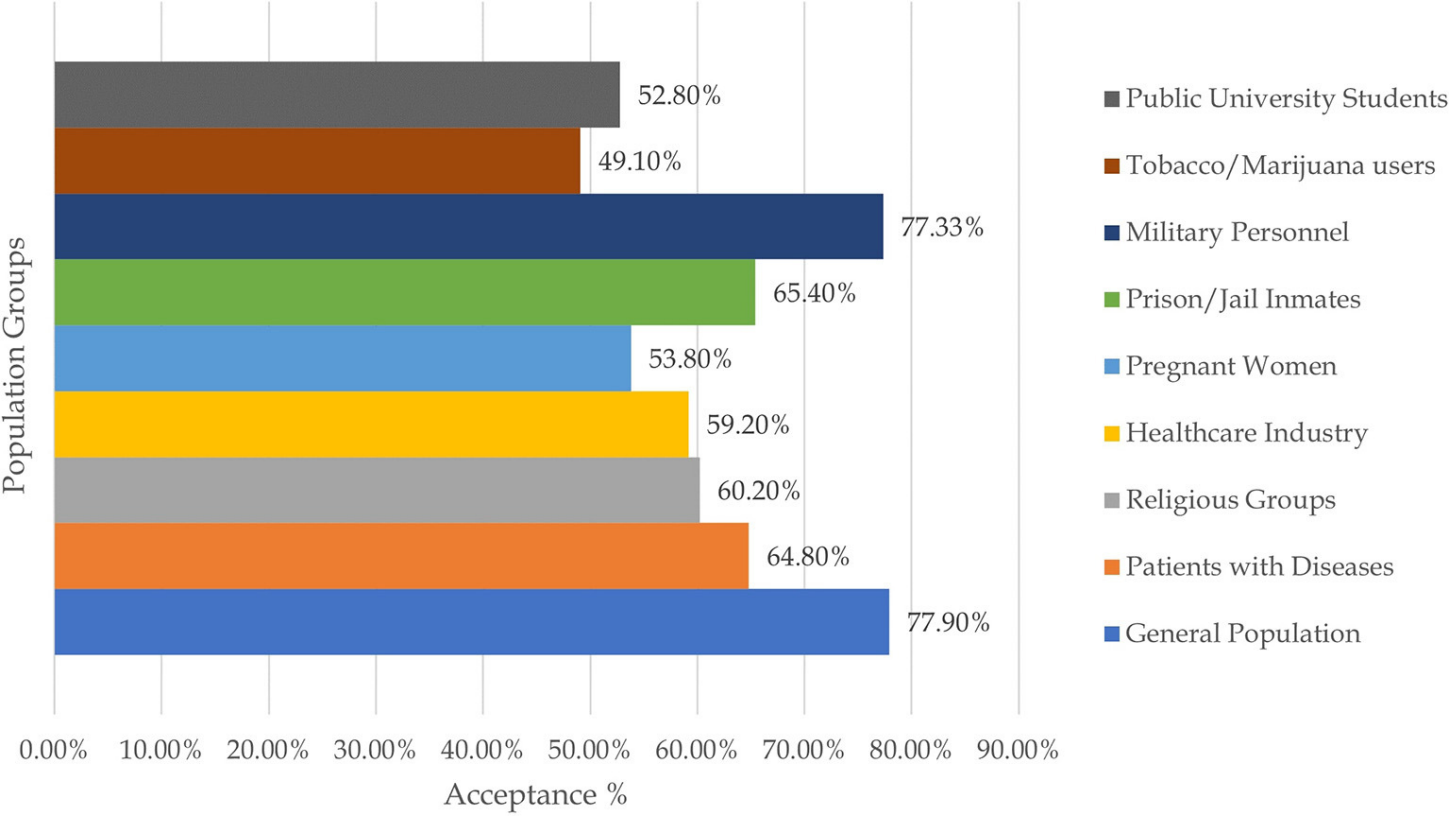
COVID-19 vaccine acceptance stratified per-respondents' population.

**Figure 4:**
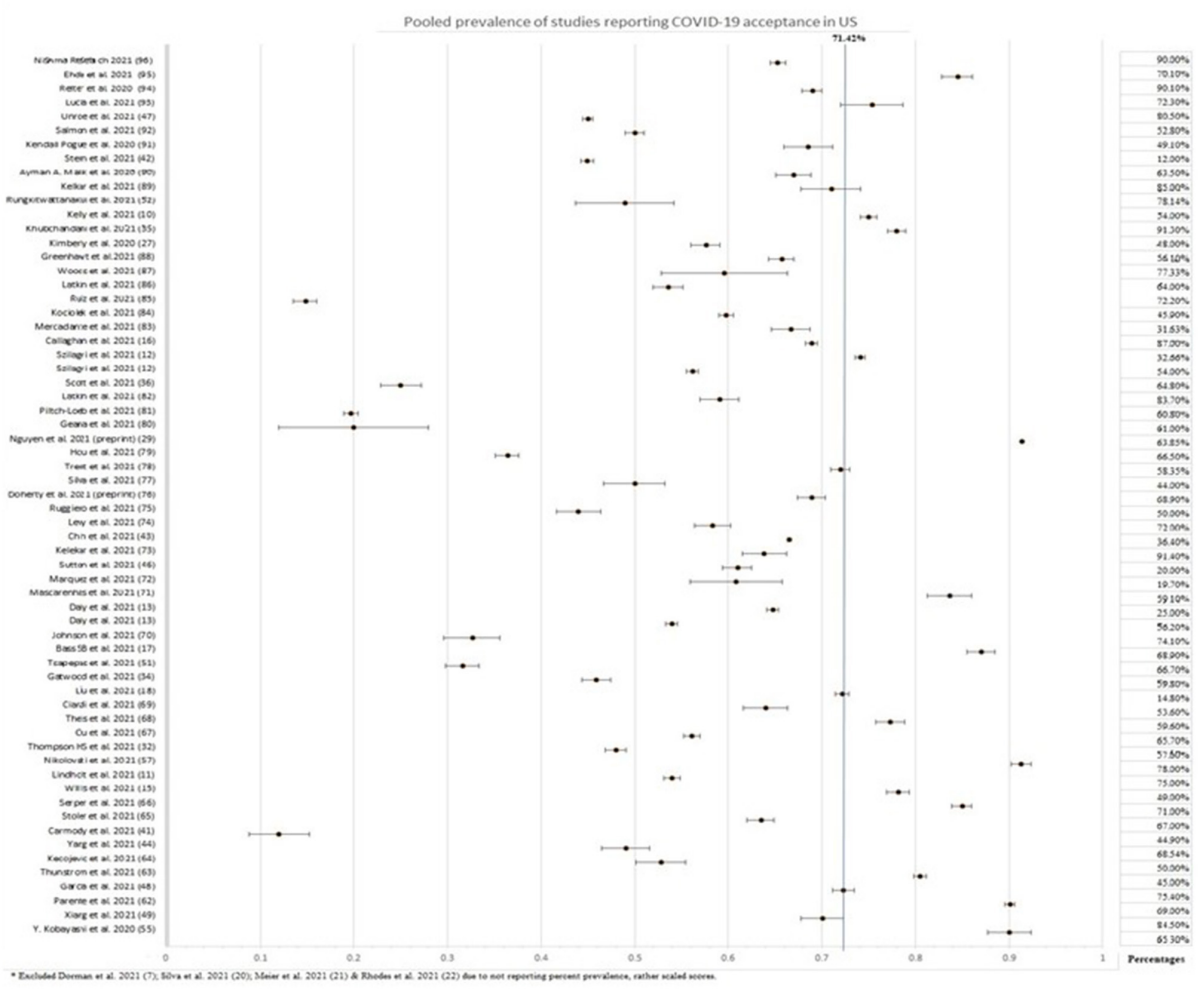
Pooled prevalence of COVID-19 vaccine acceptance in the United States.

Three surveys were carried out in religious communities, two in Jewish and one in the Amish community. The Amish community had an alarmingly low acceptance rate of 25%, whereas the Jewish people had 12 and 65.3%, with the latter being of greater significance due to the large sample size. Two surveys carried out in New York highlighted the increased hesitance toward the vaccine by pregnant women. Amongst pregnant women, an acceptance rate of 44.3 and 58.35% was reported, whereas 76.2% of non-pregnant women were inclined to take up the COVID-19 vaccine. Studies on vaccine acceptance in inmates and those discharged from prison found a 20, 44.9, and, 66.5% acceptance rate in women recently released from prison, inmates from four states, and a California state prison, respectively, with the latter being significant with a sample size of 97,779. Furthermore, a survey on tobacco and marijuana users showed a low vaccine uptake rate of 49.1%. In all four of these studies, Blacks/African Americans were correlated with increased unwillingness toward the COVID-19 vaccine. Figure 3 summarizes the percentage of vaccine acceptance in different population groups. Figure 4 demonstrates the pooled prevalence of COVID-19 vaccine acceptance in the United States with the pooled prevalence being 71.42%.

### Factors Associated With Vaccine Acceptance and Hesitance

Several factors were identified as leading to low or high vaccine acceptance. From the 65 studies, sex was mentioned in 22 surveys, race in 31, age in 16, education level in 19, and income status in 4. The male sex and individuals with a college degree or higher education were significantly associated with higher vaccine acceptance rates in all surveys reporting these variables. Furthermore, non-Hispanic Blacks were correlated with having the lowest vaccine acceptance in all 31 surveys whereas Whites and Asians showed a positive attitude toward uptake of the COVID-19 vaccination. People aged >45 years were linked with an increased approval of the vaccine compared to the younger population in 15 out of the 16 surveys. Respondents from lower-income backgrounds were less inclined to get vaccinated in all four studies. Apart from these significant predictors of behavior toward a vaccine, other factors that contributed to vaccine hesitancy included uncertainty about the vaccine safety and side effects entailing after its administration, religious reasons, and a lack of trust in the healthcare system. A comprehensive data evaluation of the studies is included in Table 1.

### Changes in COVID-19 Vaccine Acceptance Over Time

Two studies investigated the change in acceptance of the COVID-19 vaccine by surveying the population twice. Daly et al. conducted the first round of the survey in October 2020, followed by the second one in March 2021. Percentage acceptance rose by 10.8% from 54 to 64.8%. Furthermore, Szilagyi et al. carried out their surveys in November-December 2020 and April 2021 with 56.2 and 74.1% accepting the vaccine, respectively, marking a rise of 7.9%. However, an overall change in readiness for uptake of the vaccine was notable; the predictors of hesitance remained the same throughout time. African/American Blacks and females were the leading predictors of low acceptance in both studies. Daly et al. also correlated younger age and lower-income with the increased unwillingness of the COVID-19 vaccine.

## Discussion

The emergence of the B.1.617.2 (Delta) variant has put the United States on the forefront in the total number of COVID-19 cases countrywide (76). Studies have shown that a two-dose regime of any available vaccine significantly reduces a poor prognosis of COVID-19 (77). The rapid advent of efficacious vaccines for coronavirus is a projection of leaps in the field of medicine toward the goal of herd immunity. This, however, is threatened by the globally persistent but a re-emerging phenomenon of vaccine hesitancy and anti-vaccination, as experienced for vaccine-preventable diseases such as poliovirus and rubella. Despite a population of 59.9% receiving at least one dose of the COVID-19 vaccine (78), analyzing vaccine hesitancy is imperative to strategize vaccine delivery plans to procure maximum vaccine coverage and limit the spread of pathogens to the immunocompromised individuals and children who cannot be vaccinated.

In this systematic review, predictors of vaccine hesitancy and acceptance are studied across the U.S in the general population, pregnant women, immunocompromised individuals, students, healthcare workers, racial groups, and demographic characteristics. Factors associated with vaccine hesitancy are vastly different before and after the availability of vaccines. Before the roll-out of COVID-19 vaccines in mid-December 2020, major concerns across all the population groups were focused on the safety, effectiveness, and cost of the vaccine (38, 63). As the death toll peaked in the US, and with more publicly available data of vaccine trials, there was a considerable trend shift in the attitudes toward receiving a vaccination (33).

Thirty-three studies from a pool of 62 gathered vaccine acceptance rates from the general population, ranging from 91.4% (52) to a surprising low of 4.6% (7). Besides the difference in the timeline of each study, hesitancy was mainly driven by the lack of education and understanding of the process of vaccine development. As pharmaceutical companies develop multiple vaccines for the emerging strains, the expedited process of approval has raised concerns about its effectiveness (52), explaining their preferred choice of mRNA vaccines as opposed to any available vaccines (18).

Eleven studies report ethical and racial groups' unwillingness which stems from the deep-seated mistrust in the healthcare system. A study by Willis et al. conducted between July and August 2020 showed that one in every four Blacks/African Americans and Hispanics were hesitant to receive the COVID-19 vaccine, while COVID-19 vaccine hesitancy was highest among Black/African American respondents (50.00%), followed by Hispanic/Latinx respondents (19.18%), and White respondents (18.37%) (15). An online survey from March 2021 revealed a significant decline in vaccine hesitancy amongst the Black race (14). Black/African Americans have been reported to consistently depict suspicion toward the vaccine, with similar patterns observed previously with the influenza vaccines. These can be traced back to the Tuskegee Syphilis study which mishandled hundreds of Black individuals and has institutionalized racial discrimination since then (34, 79). Consistent with the findings of observational studies, Blacks/African American race were more susceptible to SARS-CoV-2 than White Americans race; if contracted, the percentage of hospitalization and mortality was significantly higher (80). A survey that studied COVID-19 vaccine acceptance in sexual and gender minority (SGM) group reported the least willingness in individuals who identified as Black; insensitivity from government representatives toward movements such as Black Lives Matter has deepened racial and economic disparity (20). Vaccine hesitancy in this minority group can be further explained by the pre-existing disparities with healthcare professionals, unavailability of healthcare services, and underrepresentation in clinical trials (7, 38, 64). Maximum vaccine coverage can be achieved by building trust in the government and medical services; Black/African Americans and Hispanic healthcare workers can contribute to foster trust in the system. As many belong to the low-income strata, health insurance can help recover the losses. Providing maximum transparency in COVID-19 trials through informed consent, ensuring maximum representation, and consistent access to healthcare during and after the pandemic is likely to improve turnout at vaccination centers. Entrusting distribution and vaccine manufacturing to businesses owned by Blacks will be a step toward positive vaccine trends. However, Blacks and Hispanics can only be freed from centuries of structural racism and classism in fields outside of medicine through consistent cultural policies across the US (34, 64, 79).

Two studies report COVID-19 vaccine acceptance percentages in religious minorities in the US; an average willingness of 25% in Amish families of the Holmes County in Ohio (56) and 12% in Jewish Orthodox individuals in NY (30). Previous studies have shown that outbreaks of measles, rubella, and poliovirus have significantly affected the Amish community in Ohio, reflecting their lack of concern regarding the severity of the disease (81). Similar to findings of previous surveys conducted for Amish families, concerns about the adverse effects of COVID-19 vaccines were recognized as a major factor for vaccine hesitancy. Other predictors of unwillingness included avoiding dependence on the government, while some conservative families were not convinced as the bishop did not levy importance to it (56, 82). Local governments in Pennsylvania, Ohio, and Indiana have set up vaccination facilities at health departments to increase turnout (30). However, an effective approach to spreading COVID-19 vaccine awareness and destigmatizing it in the Amish community will have to be in conjunction with the church leadership, and the Old Order Amish families which were unaffected by the religious doctrine (82). On the contrary, shared accommodation amongst the Jewish community in NY has increased the incidence of COVID-19, especially in the Chasidish sect. Regardless of the likelihood of contracting coronavirus, negative vaccine trends in Chasidish respondents in Brooklyn were observed (83). However, factors affecting their vaccination status; the belief that natural immunity was more beneficial, and the mistrust in physicians in the US, among other reasons put them on the priority list of strategizing distribution plans and approaches (30).

Individuals from ethnic minority groups or persons suffering from substance use disorders or mental illnesses are frequently incarcerated. The lack of stable housing, food supplies, and subpar treatment facilities delay diagnosis, which increases the risk of diseases like COVID-19 (68). Vaccine acceptance rates were relatively moderately high, ranging from 44 to 66% (45, 68) in prisons while 49% in smokers (29). Consistent with previous surveys, hesitancy in prisoners originated from health illiteracy and the perception that a COVID-19 vaccine was unnecessary (68). In individuals who have been exposed to marijuana and/or tobacco, low acceptance was governed by demographic characteristics and if they lived alone or with a family of more than 5 people. This could be explained by the notion that living alone meant no exposure to SARS-CoV-2 while living with a larger family meant greater coronavirus exposure, and hence natural immunity (29). Although California, Washington, and Texas have recognized the importance of vaccinating detained residents and have put them on the priority list, distrust in government facilities revokes any efforts made.

As experienced in the 2009 H1N1 influenza pandemic, a survey administered among reproductive-aged women from January 7, 2021, to January 29, 2021, established non-pregnant respondents to be most likely to accept vaccination, followed by breastfeeding responders with pregnant responders had the lowest vaccine acceptance (43). The lack of research of COVID-19 vaccines in pregnant women and females has raised major concerns related to its safety to the fetus, with many non-pregnant women considering disruption to the menstrual cycle and infertility a side effect (43). Future research should be geared toward pregnant and breastfeeding women who make up a large proportion of the population of the US.

Frontline healthcare workers and paramedics across the globe were the first to receive the COVID vaccine. Of the 9 studies reported in this systematic review, vaccine acceptance is one of the highest (60–90%) in most studies. Nursing staff showed the least willingness to vaccination (<50%) in multiple surveys which were conducted as vaccines were rolled out (30, 71). This results from the level of medical education in each group, and prospects about its long-term safety. The ethical duty of healthcare workers to not harm their patients makes relying on personal protective equipment insufficient and obligates them to urge staff and patients to receive the vaccine. COVID-19 poses a serious threat to immunocompromised patients, and upon hospitalization, such patients have an early mortality rate of 25% (26). In a population suffering from chronic illnesses, patients with multiple sclerosis have been included in trials, and thus show an acceptance rate between 60 and 70% (24, 74). However, skepticism regarding vaccines was greatly observed in solid organ transplant (SOT) patients (31% acceptance) (39) compared to hemodialysis patients (49% acceptance) (65). SOT patients suffer from comorbidities such as chronic kidney disease and diabetes. Immunosuppressive drugs reduce hyper inflammation in SOT patients, but if infected with SARS-COV-2, risk bacterial and fungal co-infection as innate and adaptive immunity is compromised (84). Of the fundamental 5Cs that determine vaccine coverage, decreased confidence and medical mistrust correlated with the lack of research in SOT respondents (39). Suboptimal humoral responses were recorded with two-dose regimes of COVID-19 vaccines in organ transplant recipients. Therefore, three doses of COVID-19 vaccines are being recommended to obtain recommended antibody titers (85).

The vaccine acceptance rates in the US vary by demographic characteristics, including age, geographical location, sex, and household income. While the country of origin of the vaccine has minimal contribution in encouraging or dissuading people from vaccination (23), statistics from March 2021 relay that vaccine hesitancy in the US was highest in low socioeconomic respondents belonging to households earning $50,000 or less, among those without a degree adults, or those aged 18–39 years (13). Significant generational difference in vaccine hesitancy is shown as Baby Boomers and Gen X have lower odds of vaccine hesitancy compared to Millennials (86). In a study from 18 to 64-year-old Tennessee adults, a positive response was received regarding vaccine safety, efficacy, importance, and benefit (38). In a different clinical study comprising U.S. adults aged 65 and older, 63.6% of participants reported they were very willing to receive a COVID-19 vaccine (33). This is possibly governed by the social media exposure to each generation; Gen Z and Millennials are more likely to be technologically advanced as compared to Baby Boomer and Gen X. Therefore, health information could be easily obtained from social media platforms such as Facebook and Twitter. A skewed display of factually incorrect information on social media and an individual's tendency to be vulnerable to social media's emotional appeal often becomes the gist of the decision made by a lot of individuals (87). Furthermore, a positive trend in vaccine acceptance among males vs. females (71), could be a result of a sampling bias thereof.

Medical mistrust is likely to improve if public health campaigns are run by health experts, CDC, or the WHO. Endorsements of COVID-19 vaccines by political figures is seen as politicizing a requirement of today, such as promotion of unapproved hydroxychloroquine by the US president wreaked havoc amongst the healthcare workers (88). Mandating vaccination in workplaces but also banning entry of unvaccinated individuals to public spaces may contribute as a factor of vaccine acceptance (89). Given the disproportionate willingness in the low-economic strata, efforts should be made to make the vaccine more accessible to them, by promoting health insurance. A study revealed only 35% of individuals were willing to pay $50 or more for the coronavirus vaccine. Therefore, adopting a unified policy of a no-cost follow-up after vaccination as introduced by a few states can remove financial barriers (73). The expedited process of COVID-19 vaccine testing and the cases of adverse events of a vaccine contributed significantly to vaccine hesitance. This mandates the role of media and pharmaceutical companies to deliver information that can be easily interpreted by the general population (88).

This systematic review reports a multitude of populations, and analyses percentages by regions in the US. However, the pooled percentages are subjected to spectrum bias since percentages for general population are pooled with terminally ill or marginalized groups. Percentage of COVID-19 vaccine acceptance is liable to random error as published studies were carried out at different phases of the coronavirus peak. Another limitation worth noting is that it does not provide a timeline change in the vaccine hesitancy across all groups, and thus does not record the change in vaccine enthusiasm. Lastly, vaccine acceptance percentages by states should be interpreted logically as demographic characteristics differ across the region.

## Conclusion

This study confirms that demographic characteristics played an important role in positive trends in vaccine acceptance, with the male sex showing higher acceptance of the COVID-19 vaccine. Least willingness was identified in the Black/African American population, and pregnant or breastfeeding women. Therefore, pharmaceutical companies and the US government must acknowledge the COVID-19 vaccine hesitancy that exists across the country. Increased hospitalization and adverse effects of COVID-19 in non-vaccinated individuals identify as potential threats to the economic and social stability of the country and aim to increase the divide. The willingness rates of vaccination are alarming in ethnic and sexual and gender minority groups, urging the need for outreach campaigns to prevent a COVID-19 outbreak in a susceptible population. Restoring trust in medical professionals and vaccines requires a strategical approach to overcome the racial injustice in the system, while timely implementation of vaccine delivery plans remains a challenge as new strains of coronavirus emerge as potential threats to the healthcare system.

## Data Availability Statement

The original contributions presented in the study are included in the article/Supplementary Material, further inquiries can be directed to the corresponding author/s.

## Author Contributions

FY: conception of the study, primary drafting of the work, final approval, and agreeing to the accuracy of the work. HN: conception of the study, major drafting of the work, final approval, and agreeing to the accuracy of the work. AM: conception of the study, drafting of the work, final approval, and agreeing to the accuracy of the work. UN: drafting of the work, final approval, and agreeing to the accuracy of the work. MA: conception of the study, critical revision of the work, final approval, and agreeing to the accuracy of the work. NC, ZY, BS, IU, C-YL, and AP: critical revision of the work, final approval, and agreeing to the accuracy of the work. All authors have read and approved the final manuscript.

## Conflict of Interest

The authors declare that the research was conducted in the absence of any commercial or financial relationships that could be construed as a potential conflict of interest.

## Publisher's Note

All claims expressed in this article are solely those of the authors and do not necessarily represent those of their affiliated organizations, or those of the publisher, the editors and the reviewers. Any product that may be evaluated in this article, or claim that may be made by its manufacturer, is not guaranteed or endorsed by the publisher.

## References

[1] Harrison EA Wu JW. Vaccine confidence in the time of COVID-19. Eur J Epidemiol. (2020) 35:325–30. 10.1007/s10654-020-00634-3 32318915 PMC7174145

[2] Chou WYS Budenz A. Considering emotion in COVID-19 vaccine communication: addressing vaccine hesitancy and fostering vaccine confidence. Health Commun. (2020) 35:1718–22. 10.1080/10410236.2020.1838096 33124475

[3] Different COVID-19 Vaccines. CDC. Available online at: https://www.cdc.gov/coronavirus/2019-ncov/vaccines/different-vaccines.html (accessed August 12, 2021).

[4] Thunström L Ashworth M Finnoff D Newbold SC. Hesitancy toward a COVID-19 vaccine. Ecohealth. (2021) 18:44–60. 10.1007/s10393-021-01524-0 34086129 PMC8175934

[5] Coustasse A Kimble C Maxik K. COVID-19 and vaccine hesitancy: a challenge the united states must overcome. J Ambul Care Manage. (2021) 44:71–5. 10.1097/JAC.0000000000000360 33165121

[6] Painter EM. Demographic characteristics of persons vaccinated during the first month of the COVID-19 vaccination program — United States, December 14, 2020–January 14, 2021. MMWR Morb Mortal Wkly Rep. (2021) 70:174–7. 10.15585/mmwr.mm7005e1 33539333 PMC7861480

[7] Dorman C Perera A Condon C Chau C Qian J Kalk K . Factors associated with willingness to be vaccinated against COVID-19 in a large convenience sample. J Commun Health. (2021) 46:1013–9. 10.1007/s10900-021-00987-0 33835369 PMC8033546

[8] McAteer J Yildirim I Chahroudi A. The VACCINES act: deciphering vaccine hesitancy in the time of COVID-19. Clin Infect Dis. (2020) 71:703–5. 10.1093/cid/ciaa433 32282038 PMC7184475

[9] Stolle LB Nalamasu R Pergolizzi JV Jr Magnusson P LeQuang J . Fact vs fallacy: the anti-vaccine discussion reloaded. Adv Ther. (2020) 37:4481–90. 10.1007/s12325-020-01502-y 32965654 PMC7509825

[10] Kelly BJ Southwell BG McCormack LA Bann CM MacDonald PDM Frasier AM . Predictors of willingness to get a COVID-19 vaccine in the U.S. BMC Infect Dis. (2021) 21:338. 10.1186/s12879-021-06023-9 33845781 PMC8039496

[11] Lindholt MF Jørgensen F Bor A Petersen MB. Public acceptance of COVID-19 vaccines: cross-national evidence on levels and individual-level predictors using observational data. BMJ Open. (2021) 11:e048172. 10.1136/bmjopen-2020-048172 34130963 PMC8210695

[12] Szilagyi PG Thomas K Shah MD Vizueta N Cui Y Vangala S . National trends in the US public's likelihood of getting a COVID-19 vaccine—April 1 to December 8, 2020. JAMA. (2021) 325:396–8. 10.1001/jama.2020.26419 33372943 PMC7772743

[13] Daly M Jones A Robinson E. Public trust and willingness to vaccinate against COVID-19 in the US from October 14, 2020, to March 29, 2021. JAMA. (2021) 325:2397–9. 10.1001/jama.2021.8246 34028495 PMC8145162

[14] Lin C Tu P Beitsch LM. Confidence and receptivity for COVID-19 vaccines: a rapid systematic review. Vaccines. (2020) 9:16. 10.3390/vaccines9010016 33396832 PMC7823859

[15] Willis DE Andersen JA Bryant-Moore K . COVID-19 vaccine hesitancy: Race/ethnicity, trust, and fear. Clin Transl Sci. (2021) 1–8. 10.1111/cts.13077 34213073 PMC8444681

[16] Callaghan T Moghtaderi A Lueck JA Hotez P Strych U Dor A . Correlates and disparities of intention to vaccinate against COVID-19. Soc Sci Med. (2021) 272:113638. 10.1016/j.socscimed.2020.113638 33414032 PMC7834845

[17] Bass SB Wilson-Genderson M Garcia DT Akinkugbe AA Mosavel M. SARS-CoV-2 vaccine hesitancy in a sample of us adults: role of perceived satisfaction with health, access to healthcare, and attention to COVID-19 news. Front Public Heal. (2021) 9:665724. 10.3389/fpubh.2021.665724 33996731 PMC8116504

[18] Liu T He Z Huang J Yan N Chen Q Huang F . A comparison of vaccine hesitancy of COVID-19 vaccination in China and the United States. Vaccines. (2021) 9:649. 10.3390/vaccines9060649 34198716 PMC8232230

[19] Page MJ McKenzie JE Bossuyt PM Boutron I Hoffmann TC Mulrow CD . The PRISMA 2020 statement: an updated guideline for reporting systematic reviews. BMJ. (2021) 372:n71. 10.1136/bmj.n71 33782057 PMC8005924

[20] Silva DT da Biello K Lin WY Valente PK Mayer KH Hightow-Weidman L . COVID-19 vaccine acceptance among an online sample of sexual and gender minority men and transgender women. Vaccines. (2021) 9:1–10. 10.3390/vaccines9030204 33804530 PMC7999863

[21] Meier BP Dillard AJ Lappas CM. Predictors of the intention to receive a SARS-CoV-2 vaccine. J Public Health. (2021) fdab013. 10.1093/pubmed/fdab013 33677601 PMC7989339

[22] Rhodes ME Sundstrom B Ritter E McKeever BW McKeever R. Preparing for a COVID-19 vaccine: a mixed methods study of vaccine hesitant parents. J Health Commun. (2020) 25:831–7. 10.1080/10810730.2021.1871986 33719886

[23] Kobayashi Y Howell C Heinrich T. Vaccine hesitancy, state bias, and Covid-19: evidence from a survey experiment using Phase-3 results announcement by BioNTech and Pfizer. Soc Sci Med. (2021) 282:114115. 10.1016/j.socscimed.2021.114115 34157613 PMC8205290

[24] Xiang XM Hollen C Yang Q Brumbach BH Spain RI Wooliscroft L. COVID-19 vaccination willingness among people with multiple sclerosis. Mult Scler J Exp Transl Clin. (2021) 7. 10.1177/20552173211017159 34104472 PMC8172949

[25] Parente DJ Ojo A Gurley T Le Master JW Meyer M Wild DM . Acceptance of COVID-19 vaccination among health system personnel. J Am Board Fam Med. (2021) 34:498–522. 10.3122/jabfm.2021.03.200541 34088810

[26] Garcia P Montez-Rath ME Moore H Flotte J Fults C Block MS . SARS-CoV-2 vaccine acceptability in patients on hemodialysis: a nationwide survey. J Am Soc Nephrol. (2021) 32:1575–81. 10.1681/ASN.2021010104 33927004 PMC8425649

[27] Thunstrom L Ashworth M Finnoff D Newbold S. Hesitancy towards a COVID-19 vaccine and prospects for herd immunity. SSRN Electron J. (2020) 10.2139/ssrn.3593098 PMC8175934 34086129

[28] Kecojevic A Basch CH Sullivan M Chen YT Davi NK. COVID-19 vaccination and intention to vaccinate among a sample of college students in New Jersey. J Commun Health. (2021) 11:179–85. 10.1007/s10900-021-00992-3 33905034 PMC8077859

[29] Yang Y Dobalian A Ward KD. COVID-19 vaccine hesitancy and its determinants among adults with a history of tobacco or marijuana use. J Commun Health. (2021) 1–9. 10.1007/s10900-021-00993-2 33956270 PMC8101333

[30] Carmody ER Zander D Klein EJ Mulligan MJ Caplan AL. Knowledge and attitudes toward covid-19 and vaccines among a New York Haredi-Orthodox Jewish Community. J Commun Health. (2021) 1–9. 10.1007/s10900-021-00995-0 33999317 PMC8127857

[31] Stoler J Enders AM Klofstad CA Uscinski JE. The limits of medical trust in mitigating COVID-19 vaccine hesitancy among black Americans. J Gen Intern Med. (2021) 1–3. 10.1007/s11606-021-06743-3 34021446 PMC8139367

[32] Serper M Reddy KR Bewtra M Ahmad N Mehta SJ. COVID-19 vaccine perceptions among patients with chronic disease in a large gastroenterology and hepatology practice. Am J Gastroenterol. (2021) 116:1345–9. 10.14309/ajg.0000000000001270 33878043 PMC8172451

[33] Nikolovski J Koldijk M Weverling GJ Spertus J Turakhia M Saxon L . Factors indicating intention to vaccinate with a COVID-19 vaccine among older U.S. adults. PLoS ONE. (2021) 16:e0251963. 10.1371/journal.pone.0251963 34029345 PMC8143399

[34] Thompson HS Manning M Mitchell J Kim S Harper FWK Cresswell S . Factors associated with racial/ethnic group–based medical mistrust and perspectives on COVID-19 vaccine trial participation and vaccine uptake in the US. JAMA Netw Open. (2021) 4:e2111629. 10.1001/jamanetworkopen.2021.11629 34042990 PMC8160590

[35] Ou MT Boyarsky BJ Zeiser LB Chiang TP-Y Ruddy J Rasmussen SEVP . Kidney transplant recipient attitudes toward a SARS-CoV-2 vaccine. Transplant Direct. (2021) 7:e713. 10.1097/TXD.0000000000001171 34131585 PMC8196090

[36] Theis SR Li PC Kelly D Ocampo T Berglund A Morgan D . Perceptions and concerns regarding COVID-19 vaccination in a military base population. Mil Med. (2021) 2021:usab230. 10.1093/milmed/usab230 34117501 PMC8344492

[37] Ciardi F Menon V Jensen JL Shariff MA Pillai A Venugopal U . Knowledge, attitudes and perceptions of COVID-19 vaccination among healthcare workers of an inner-city hospital in New York. Vaccines. (2021) 9:516. 10.3390/vaccines9050516 34067743 PMC8156250

[38] Gatwood J McKnight M Fiscus M Hohmeier KC Chisholm-Burns M. Factors influencing likelihood of COVID-19 vaccination: a survey of Tennessee adults. Am J Health Syst Pharm. (2021) 78:879–89. 10.1093/ajhp/zxab099 33954426 PMC7989652

[39] Tsapepas D Husain SA King KL Burgos Y Cohen DJ Mohan S. Perspectives on COVID-19 vaccination among kidney and pancreas transplant recipients living in New York City. Am J Heal Pharm. (2021) zxab272. 10.1093/ajhp/zxab272 34185824 PMC8344809

[40] Johnson KD Akingbola O Anderson J Hart J Chapple A Woods C . Combatting a “Twin-demic”: a quantitative assessment of COVID-19 and influenza vaccine hesitancy in primary care patients. Heal Promot Perspect. (2021) 11:179–85. 10.34172/hpp.2021.22 34195041 PMC8233667

[41] Mascarenhas AK Lucia VC Kelekar A Afonso NM. Dental students' attitudes and hesitancy toward COVID-19 vaccine. J Dent Educ. (2021) 85:1504–10. 10.1002/jdd.12632 33913152 PMC8242421

[42] Marquez RR Gosnell ES Thikkurissy S Schwartz SB Cully JL. Caregiver acceptance of an anticipated COVID-19 vaccination. J Am Dent Assoc. (2021) 152:730–9. 10.1016/j.adaj.2021.03.004 34059293 PMC7988472

[43] Sutton D D'Alton M Zhang Y Kahe K Cepin A Goffman D . COVID-19 vaccine acceptance among pregnant, breastfeeding, and nonpregnant reproductive-aged women. Am J Obstet Gynecol MFM. (2021) 3:100403. 10.1016/j.ajogmf.2021.100403 34048965 PMC8146275

[44] Kelekar AK Lucia VC Afonso NM Mascarenhas AK. COVID-19 vaccine acceptance and hesitancy among dental and medical students. J Am Dent Assoc. (2021) 152:596–603. 10.1016/j.adaj.2021.03.006 34030867 PMC7997309

[45] Chin ET Leidner D Ryckman T Liu YE Prince L Alarid-Escudero F . Covid-19 vaccine acceptance in california state prisons. N Engl J Med. (2021) 385:374–6. 10.1056/NEJMc2105282 33979505 PMC8133697

[46] Levy AT Singh S Riley LE Prabhu M. Acceptance of COVID-19 vaccination in pregnancy: a survey study. Am J Obstet Gynecol MFM. (2021) 3:100399. 10.1016/j.ajogmf.2021.100399 34020098 PMC8129996

[47] Ruggiero KM Wong J Sweeney CF Avola A Auger A Macaluso M . Parents' intentions to vaccinate their children against COVID-19. J Pediatr Heal Care. (2021) 35:509–17. 10.1016/j.pedhc.2021.04.005 34217553 PMC8245313

[48] Doherty IA Pilkington W Brown L Billings V Hoffler U Paulin L . COVID-19 vaccine hesitancy in underserved communities of North Carolina. medRxiv. (2021). 10.1101/2021.02.21.21252163 34723973 PMC8559933

[49] Silva J Bratberg J Lemay V. COVID-19 and influenza vaccine hesitancy among college students. J Am Pharm Assoc. (2021). 10.1016/j.japh.2021.05.009 34092517 PMC8139529

[50] Trent M Seale H Ahmad Chughtai A Salmon D Raina MacIntyre C. Trust in government, intention to vaccinate and COVID-19 vaccine hesitancy: a comparative survey of five large cities in the United States, United Kingdom, and Australia. Vaccine. (2021). 10.1016/j.vaccine.2021.06.048 34218963 PMC8220944

[51] Hou Z Tong Y Du F Lu L Zhao S Yu K . Assessing covid-19 vaccine hesitancy, confidence, and public engagement:a global social listening study. J Med Internet Res. (2021) 23:e27632. 10.2196/27632 34061757 PMC8202656

[52] Nguyen LH Joshi AD Drew DA Merino J Ma W Lo C-H . Racial and ethnic differences in COVID-19 vaccine hesitancy and uptake. medRxiv. (2021). 10.1101/2021.02.25.21252402 33655271 PMC7924296

[53] Geana M V Anderson S Ramaswamy M. COVID-19 vaccine hesitancy among women leaving jails: a qualitative study. Public Health Nurs. (2021) 38:892–96. 10.1111/phn.12922 33973268 PMC8242643

[54] Piltch-Loeb R Savoia E Goldberg B Hughes B Verhey T Kayyem J . Examining the effect of information channel on COVID-19 vaccine acceptance. PLoS ONE. (2021) 16:e0251095. 10.1371/journal.pone.0251095 33979370 PMC8116041

[55] Latkin C Dayton LA Yi G Konstantopoulos A Park J Maulsby C . COVID-19 vaccine intentions in the United States, a social-ecological framework. Vaccine. (2021) 39:2288. 10.1016/j.vaccine.2021.02.058 33771392 PMC7945864

[56] Scott EM Stein R Brown MF Hershberger J Scott EM Wenger OK. Vaccination patterns of the northeast Ohio Amish revisited. Vaccine. (2021) 39:1058–63. 10.1016/j.vaccine.2021.01.022 33478791

[57] Mercadante AR Law A V. Will they, or Won't they? Examining patients' vaccine intention for flu and COVID-19 using the Health Belief Model. Res Soc Adm Pharm. (2021) 17:1596. 10.1016/j.sapharm.2020.12.012 33431259 PMC7833824

[58] Kociolek LK Elhadary J Jhaveri R Patel AB Stahulak B Cartland J. Coronavirus disease 2019 vaccine hesitancy among children's hospital staff: a single-center survey. Infect Control Hosp Epidemiol. (2021) 42:775–7. 10.1017/ice.2021.58 33557977 PMC7925985

[59] Ruiz JB Bell RA. Predictors of intention to vaccinate against COVID-19: results of a nationwide survey. Vaccine. (2021) 39:1080–6. 10.1016/j.vaccine.2021.01.010 33461833 PMC7794597

[60] Latkin CA Dayton L Yi G Colon B Kong X. Mask usage, social distancing, racial, and gender correlates of COVID-19 vaccine intentions among adults in the US. PLoS ONE. (2021) 16:e0246970. 10.1371/journal.pone.0246970 33592035 PMC7886161

[61] Keene Woods N Vargas I McCray-Miller M Drassen Ham A Chesser AK. SARS-CoV2, the COVID-19 pandemic and community perceptions. J Prim Care Community Heal. (2021) 12. 10.1177/2150132721995451 33596683 PMC7897804

[62] Greenhawt M Kimball S DunnGalvin A Abrams EM Shaker MS Mosnaim G . Media influence on anxiety, health utility, and health beliefs early in the SARS-CoV-2 pandemic—a survey study. J Gen Intern Med. (2021) 36:1327. 10.1007/s11606-020-06554-y 33629267 PMC7904294

[63] Fisher KA Bloomstone SJ Walder J Crawford S Fouayzi H Mazor KM. Attitudes toward a potential SARS-CoV-2 vaccine : a survey of U.S. adults. Ann Intern Med. (2020) 173:964–73. 10.7326/M20-3569 32886525 PMC7505019

[64] Khubchandani J Sharma S Price JH Wiblishauser MJ Sharma M Webb FJ. COVID-19 vaccination hesitancy in the united states: a rapid national assessment. J Community Health. (2021) 46:270–7. 10.1007/s10900-020-00958-x 33389421 PMC7778842

[65] Rungkitwattanakul D Yabusaki A Singh D Lawson P Nwaogwugwu U Iheagwara OS . COVID-19 vaccine hesitancy among African American hemodialysis patients: a single-center experience. Hemodial Int. (2021) 25:410–2. 10.1111/hdi.12922 33709553 PMC8250524

[66] Kelkar AH Blake JA Cherabuddi K Cornett H McKee BL Cogle CR. Vaccine enthusiasm and hesitancy in cancer patients and the impact of a webinar. Healthc. (2021) 9:351. 10.3390/healthcare9030351 33808758 PMC8003419

[67] Malik AA McFadden SAM Elharake J Omer SB. Determinants of COVID-19 vaccine acceptance in the US. EClinicalMedicine. (2020) 26:100495. 10.1016/j.eclinm.2020.100495 32838242 PMC7423333

[68] Stern MF Piasecki AM Strick LB Rajeshwar P Tyagi E Dolovich S . Willingness to receive a COVID-19 vaccination among incarcerated or detained persons in correctional and detention facilities — four states, September–December (2020). MMWR Morb Mortal Wkly Rep. (2021) 70:473–7. 10.15585/mmwr.mm7013a3 33793457 PMC8022882

[69] Pogue K Jensen JL Stancil CK Ferguson DG Hughes SJ Mello EJ . Influences on attitudes regarding potential covid-19 vaccination in the united states. Vaccines. (2020) 8:1–14. 10.3390/vaccines8040582 33022917 PMC7711655

[70] Salmon DA Dudley MZ Brewer J Kan L Gerber JE Budigan H . COVID-19 vaccination attitudes, values and intentions among United States adults prior to emergency use authorization. Vaccine. (2021) 39:2698–711. 10.1016/j.vaccine.2021.03.034 33781601 PMC7988387

[71] Unroe KT Evans R Weaver L Rusyniak D Blackburn J. Willingness of long-term care staff to receive a COVID-19 vaccine: a single state survey. J Am Geriatr Soc. (2021) 69:593–9. 10.1111/jgs.17022 33370448

[72] Lucia VC Kelekar A Afonso NM. COVID-19 vaccine hesitancy among medical students. J Public Health. (2020) 43:445–9. 10.1093/pubmed/fdaa230 33367857 PMC7799040

[73] Reiter PL Pennell ML Katz ML. Acceptability of a COVID-19 vaccine among adults in the United States: how many people would get vaccinated? Vaccine. (2020) 38:6500–7. 10.1016/j.vaccine.2020.08.043 32863069 PMC7440153

[74] Ehde DM Roberts MK Herring TE Alschuler KN. Willingness to obtain COVID-19 vaccination in adults with multiple sclerosis in the United States. Mult Scler Relat Disord. (2021) 49:102788. 10.1016/j.msard.2021.102788 33508570 PMC7825851

[75] Nishma Research. COVID-19 Attitudes Vaccine Sentiment in the U.S. Orthodox Jewish Community: Views Among the Chasidish, Yeshivish, and Modern Orthodox Segments. (2021). Available online at: http://nishmaresearch.com (accessed August 23, 2021).

[76] Coronavirus Cases Worldwide by Country. Statista. Available online at: https://www.statista.com/statistics/1043366/novel-coronavirus-2019ncov-cases-worldwide-by-country/ (accessed August 16, 2021).

[77] Bernal JL Andrews N Gower C Gallagher E Simmons R Thelwall S . Effectiveness of Covid-19 vaccines against the B.1.617.2 (Delta) variant. N Engl J Med. (2021) 385:585–94. 10.1056/NEJMoa2108891 34289274 PMC8314739

[78] U.S. COVID-19 Vaccine Tracker: See Your State's Progress. Mayo Clinic. Available online at: https://www.mayoclinic.org/coronavirus-covid-19/vaccine-tracker (accessed August 17, 2021).

[79] Warren RC Forrow L Hodge DA Sr Truog RD. Trustworthiness before trust - covid-19 vaccine trials and the black community. N Engl J Med. (2020) 383:e121. 10.1056/NEJMp2030033 33064382

[80] Vahidy FS Mph M Carlos J Bs N Meeks JR Khan Bs O . Racial and ethnic disparities in SARS-CoV-2 pandemic: analysis of a COVID-19 observational registry for a diverse U.S. metropolitan population. BMJ Open. (2020) 10:e039849. 10.1136/bmjopen-2020-039849 32784264 PMC7418666

[81] Yoder JS Dworkin MS. Vaccination usage among an old-order Amish community in Illinois. Pediatr Infect Dis J. (2006) 25:1182–3. 10.1097/01.inf.0000246851.19000.3e 17133167

[82] Kettunen C Nemecek J Wenger O. Evaluation of low immunization coverage among the Amish population in rural Ohio. AJIC Am J Infect Control. (2017) 45:630–4. 10.1016/j.ajic.2017.01.032 28302434

[83] Nishma Research. COVID-19 Attitudes Vaccine Sentiment in the U.S. Orthodox Jewish Community: Views Among the Chasidish, Yeshivish, and Modern Orthodox Segment. (2021). Available online at: http://nishmaresearch.com (accessed August 18, 2021).

[84] Ao G Wang Y Qi X Nasr B Bao M Gao M . The association between severe or death COVID-19 and solid organ transplantation: a systematic review and meta-analysis. Transplant Rev. (2021) 35:100628. 10.1016/j.trre.2021.100628 34087553 PMC8137345

[85] Werbel WA Boyarsky BJ Ou MT Massie AB Tobian AAR Garonzik-Wang JM . Safety and immunogenicity of a third dose of SARS-CoV-2 vaccine in solid organ transplant recipients: a case series. Ann Intern Med. (2021) 174:1330–2. 10.7326/L21-0282 34125572 PMC8252023

[86] Shih S-F Wagner AL Masters NB Prosser LA Lu Y Zikmund-Fisher BJ. Vaccine hesitancy and rejection of a vaccine for the novel coronavirus in the United States. Front Immunol. (2021). 12:558270. 10.3389/fimmu.2021.558270 34194418 PMC8236639

[87] N P EA C H H K G. Social media and vaccine hesitancy: new updates for the era of COVID-19 and globalized infectious diseases. Hum Vaccin Immunother. (2020) 16:2586–93. 10.1080/21645515.2020.1780846 32693678 PMC7733887

[88] Bunch L. A tale of two crises: addressing covid-19 vaccine hesitancy as promoting racial justice. Hec Forum. (2021) 33:143–54. 10.1007/s10730-021-09440-0 33464452 PMC7814857

[89] Vergara RJD Sarmiento PJD Lagman JDN. Building public trust: a response to COVID-19 vaccine hesitancy predicament. J Public Health. (2021) 43:e291–2. 10.1093/pubmed/fdaa282 33454769 PMC7928772

