# Supplementary material for: COVID-19 Vaccine Hesitancy in the United States: A Systematic Review
Source: Front Public Health. 2021 Nov 23;9:770985. doi: 10.3389/fpubh.2021.770985 (PMC8650625; doi:10.3389/fpubh.2021.770985)
Supplement: Supplementary file 1 [file Data_Sheet_1.docx]

| **State** | **Vaccine Acceptance %** |
| --- | --- |
| Alabama | 0 |
| Arizona | 76 |
| Arkansas | 78.14 |
| California | 66.5 |
| Colorado | 0 |
| Connecticut | 0 |
| Delaware | 0 |
| District of Columbia | 0 |
| Florida | 54.3 |
| Georgia | 0 |
| Idaho | 0 |
| Illinois | 59.8 |
| Indiana | 45 |
| Iowa | 0 |
| Kansas | 89.6 |
| Kentucky | 0 |
| Louisiana | 32.66 |
| Maine | 0 |
| Maryland | 0 |
| Massachusetts | 0 |
| Michigan | 58.7 |
| Minnesota | 0 |
| Mississippi | 0 |
| Missouri | 0 |
| Montana | 0 |
| Nebraska | 0 |
| Nevada | 0 |
| New Hampshire | 0 |
| New Jersey | 52.5 |
| New Mexico | 0 |
| New York | 58.5 |
| North Carolina | 68.9 |
| North Dakota | 0 |
| Ohio | 60.4 |
| Oklahoma | 0 |
| Oregon | 70.1 |
| Pennsylvania | 85 |
| Rhode Island | 50 |
| South Carolina | 0 |
| South Dakota | 0 |
| Tennessee | 45.9 |
| Texas | 45.5 |
| Utah | 80.3 |
| Vermont | 0 |
| Virginia | 0 |
| Washington | 43.5 |
| West Virginia | 0 |
| Wisconsin | 0 |
| Wyoming | 0 |

**Online Supplementary Material**

**Supplementary Table 1: Average acceptance % of COVID-19 vaccine by states**

| **PubMed** | (COVID∗ vaccine∗ hesitancy [Title/Abstract]) OR (COVID∗ vaccine acceptance [Title/Abstract])) OR (COVID∗ vaccine∗ hesitancy [Title/Abstract])) OR (COVID ∗ intention to vaccinate∗ [Title/Abstract]) OR (COVID vaccine∗accept∗[Title/Abstract]) |
| --- | --- |
| **Cochrane Central** | (COVID vaccine hesitancy OR COVID vaccine acceptance OR COVID vaccine hesitancy OR COVID intention to vaccinate OR COVID vaccine accept*) |

**Supplementary Table 2: Detailed search strategy**
